# Mild and Efficient Strontium Chloride Hexahydrate-Catalyzed Conversion of Ketones and Aldehydes into Corresponding *gem*- Dihydroperoxides by Aqueous H_2_O_2_

**DOI:** 10.3390/molecules15031433

**Published:** 2010-03-08

**Authors:** Davood Azarifar, Kaveh Khosravi, Fatemeh Soleimanei

**Affiliations:** Faculty of Chemistry, Bu-Ali Sina University, 65178 Hamadan, Iran; E-Mails: k_khosravi@basu.ac.ir (K.K.); f.soleimani61@yahoo.com (F.S.)

**Keywords:** *gem*-dihydroperoxide, strontium chloride hexahydrate, ketone, aldehyde, hydrogen peroxide

## Abstract

SrCl_2_·6H_2_O has been shown to act as an efficient catalyst for the conversion of aldehydes or ketones into the corresponding *gem*-dihydroperoxides (DHPs) by treatment with aqueous H_2_O_2_ (30%) in acetonitrile. The reactions proceed under mild and neutral conditions at room temperature to afford good to excellent yields of product.

## 1. Introduction

In recent years, much research has been directed towards *gem*-dihydroperoxides (DHPs) [1], due to their importance as useful intermediates in the synthesis of various peroxides, including tetraoxanes [2,3,4,5,6,7,8,9], and their analogues such as silatetroxanes [10], spirobisperoxyketals [11], and tetroxycycloalkanes [12], and epoxidation of α,β-unsaturated ketones [13]. These compounds have also recently been utilized as effective reagents in: (i) oxidation of various compounds [14] such as sulfides [15], (ii) enantioselective oxidation of 2-substituted 1,4-naphthoquinones [16], and (iii) as initiators in polymerization reactions [17,18]. It is also remarkable that *gem*-dihydroperoxides are relevant to peroxidic antimalarial drugs [2,19,20,21,22,23] possessing the *gem*-peroxy linkage as a salient structural feature [23,24,25,26] in common with many well-known antimalarial cyclic organic peroxides [1,2,27,28,29,30,31,32,33,34,35]. Most of the documented protocols for the synthesis of *gem*-dihydroperoxides in the literature suffer from significant drawbacks such as the use of strong acidic media, concentrated H_2_O_2_ and low yields [1]. These methods mainly utilize a Brönsted or Lewis acid e.g., HCO_2_H [12,20,36], NaHSO_4_-SiO_2 _ [37], H_2_SO_4_ [38], F_3_CCO_2_H [39], H_2_WO_4_ [29,38], and BF_3_·OEt_2 _[30,39] to promote the conversion of ketones, ketals or enol ethers into the corresponding DHPs on treatment with aqueous H_2_O_2_. Other catalysts such as methyltrioxorhenium (prepared from Re_2_O_7_) [2], ceric ammonium nitrate (CAN) [32], and iodine [33] have also been reported to promote such transformations. However, these methods are not mild enough to offer general applicability and have limitations such as low yields, long reaction times, use of high concentration of H_2_O_2_ and incompatibility with sensitive functional groups. Recently, Dussault has reported a remarkably mild and highly efficient protocol for Re_2_O_7_-catalyzed conversion of ketones, aldehydes or acetals into 1,1-dihydroperoxides by H_2_O_2_ which represents a major improvement [34].

## 2. Results and Discussion

As part of our ongoing efforts to develop new methods for the synthesis of DHPs, we report here another new and highly efficient and inexpensive catalyst SrCl_2_·6H_2_O to promote the synthesis of *gem*-DHPs from ketones and aldehydes employing aqueous H_2_O_2_ (30%) at room temperature. To achieve suitable reaction conditions, *i.e.*, lower reaction times and higher yields, for the conversion of the ketones and aldehydes into their corresponding DHPs, various Lewis acid catalysts and solvents were investigated using 3-pentanone, cyclohexanone, acetophenone, and benzaldehyde as test compounds at room temperature (Table 1). 

**Table 1:** Effects of catalyst and solvent in the synthesis of *gem*-DHPs.^ a^

Entry	Ketone 1/Aldehyde 3	Catalyst	Solvent	Time (h)	Yield (%)^b^
1	3-pentanone	SrCl_3_·6H_2_O	CH_3_CN	3	95
2	3-pentanone	SrCl_3_·6H_2_O	CH_2_Cl_2_	6	78
3	3-pentanone	SrCl_3_·6H_2_O	Et_2_O	8	56
4	3-pentanone	SrCl_3_·6H_2_O	AcOEt	6	82
5	3-pentanone	SbCl_3_	CH_3_CN	8	48
6	3-pentanone	CeO_2_	CH_3_CN	10	45
7	3-pentanone	CrCl_3_·6H_2_O	CH_3_CN	8	75
8	3-pentanone	KF-Al_2_O_3_	CH_3_CN	10	Trace
9	Cyclohexanone	SrCl_3_·6H_2_O	CH_3_CN	3	94
10	Cyclohexanone	SbCl_3_	CH_3_CN	7	55
11	Cyclohexanone	CeO_2_	CH_3_CN	8	50
12	Cyclohexanone	CrCl_3_·6H_2_O	CH_3_CN	6	70
13	Cyclohexanone	KF-Al_2_O_3_	CH_3_CN	10	Trace
14	Acetophenone	SrCl_3_·6H_2_O	CH_3_CN	10	45
15	Acetophenone	SbCl_3_	CH_3_CN	12	23
16	Acetophenone	CeO_2_	CH_3_CN	12	15
17	Acetophenone	CrCl_3_·6H_2_O	CH_3_CN	10	28
18	Acetophenone	KF-Al_2_O_3_	CH_3_CN	20	0
19	Benzaldehyde	SrCl_3_·6H_2_O	CH_3_CN	10	54
20	Benzaldehyde	SbCl_3_	CH_3_CN	15	32
21	Benzaldehyde	CeO_2_	CH_3_CN	15	15
22	Benzaldehyde	CrCl_3_·6H_2_O	CH_3_CN	12	22
23	Benzaldehyde	KF-Al_2_O_3_	CH_3_CN	20	0

^a^ *Conditions*: Ketone and aldehyde (1 mmol), solvent (4 mL), catalyst (0.1 mmol), 30% aq. H_2_O_2 _(3 mL), reactions are carried out at rt. ^b ^Isolated yields.

As can be seen in Table 1, the reaction worked best in terms of yield and reaction time with aqueous H_2_O_2_ (30%) when SrCl_2_·6H_2_O (10 mol %) was used as a catalyst. The other catalysts such as SbCl_3_, CeO_2_ and CrCl_3_·6H_2_O gave moderate to low yields while KF-Al_2_O_3 _was found to be completely unsuitable for the synthesis of these DHPs. Effects of the solvents such as CH_2_Cl_2_, Et_2_O, MeCN and AcOEt on the yields of the products were tested and the results are summarized in Table 1. Acetonitrile appeared as a much better solvent compared with other ones. This suggests that solvent polarity plays an important role in the synthesis of DHPs.

**Scheme 1:**
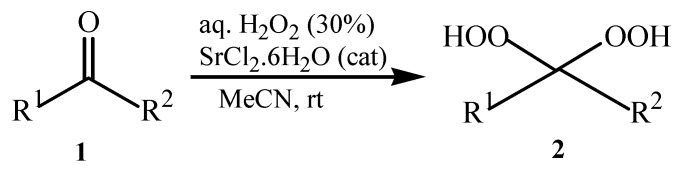
Synthesis of *gem*-dihydroperoxides **2a-g.**

This success encouraged us to extend these reaction conditions to a variety of cyclic and acyclic aliphatic ketones **1a-g **using aqueous H_2_O_2_ (30%) in the presence of 10 mol% amount of SrCl_2_·6H_2_O as a chosen catalyst in acetonitrile at room temperature. The corresponding *gem*-dihydroperoxides **2a-g **were produced in high to excellent yields (90–98%) within 3–12 h (Table 2, Scheme 1). Similarly, aromatic ketones **1h-j **and aromatic aldehydes **1l-p **were converted to their corresponding *gem*-DHPs **2h-j** and **2l-p** in (45–68%) and (52–75%) yields respectively (Table 1). However, under the same reaction condition no conversion to *gem*-DHP was observed for benzophenone **1k **and it was recovered almost intact after 12 hours. This can possibly be accounted for by the strong resonance stabilization and steric effects exerted by two phenyl groups.

**Scheme 2:**
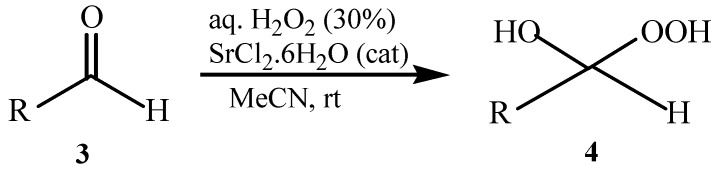
Syntheiss of hydroxyl-hydroperoxides **4q** and **4r.**

As previously reported by Rieche [40] and Žmitek *et al.* [41], we observed in the present protocol that simple, nonaromatic aldehydes such as octanal **3q **and dihydrocinnamaldehyde **3r**, which easily undergo hydration [42], reacted differently from the ketones and aromatic aldehydes. Under the same reaction conditions which converted ketones and aromatic aldehydes into their corresponding DHPs, both alkyl aldehydes-octanal **3q **and dihydrocinnamaldehyde **3r**-were not converted into their corresponding DHPs but instead into hydroxyl-hydroperoxides **4q** and **4r** in high yields (Table 1, Scheme 2), that is the addition of only one molecule of hydrogen peroxide to the carbonyl group has occurred. This implies that our protocol can furnish another hitherto unreported approach to 1,1-hydroxyhydroperoxides from aliphatic aldehydes.

**Table 2:** Synthesis of *gem*-dihydroperoxides with SrCl_2_·6H_2_O (cat.)/30% aq. H_2_O_2._^ a^

Entry	Ketone 1/ Aldehyde 3	Product2 or 4^ b^	Time (h)	Yield (%)^c^
**a**	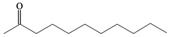	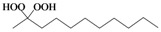	4	96
**b**	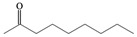	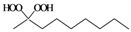	4	98
**c**	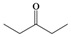	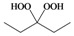	3	95
**d**	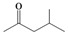	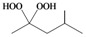	3	92
**e**	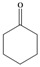	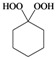	3	94
**f**	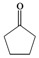	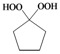	4	97
**g**	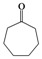	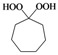	3	90
**h**	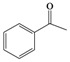	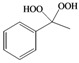	10	45
**i**	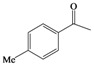	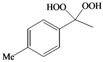	9	68
**j**	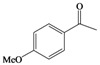	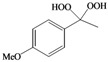	8	62
**k**	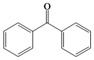	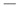	12	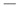
**l**	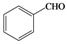	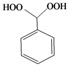	10	54
**m**	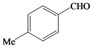	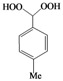	11	52
**n**	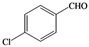	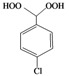	9	75
**o**	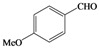	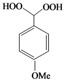	9	72
**P^d^**	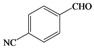	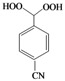	10	73
**q**	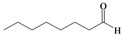	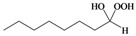	5	90
**r**	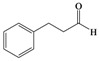	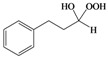	5	92

^a^ *Conditions*: Ketone and aldehyde (1 mmol), acetonitrile (4 mL), SrCl_2_·6H_2_O (0.1 mmol), 30% aq. H_2_O_2 _(3 mL), reactions are carried out at rt. ^b ^The structures of the products were established from their physical properties and spectral (^1^H-, ^13^C-NMR and MS) analysis and compared with the literature. ^c^ Isolated Yield. ^d ^A new derivative.

## 3. Experimental

### 3.1. General

Chemicals were obtained from Merck. FT-IR spectra were recorded on a Shimadzu 435-U-04 spectrophotometer (KBr pellets). ^1^H- and ^13^C-NMR spectra were recorded on a 200 (50) MHz Varian or JEOL FX 90 MHz spectrometers in CDCl_3_ and DMSO-d_6_ solution, and are reported in δ units with TMS as internal standard. Melting points were determined in open capillary tubes in a Stuart SMP_3_ apparatus and uncorrected. 

### 3.2. General procedure for synthesis of gem-dihydroperoxides

*Caution*: Peroxidic compounds are potentially explosive and require precautions in handling (shields, fume hoods, absence of transition metal salts and heating).

A mixture of carbonyl substrates **1** or **3** (1 mmol), 30% aqueous H_2_O_2_ (3 mL) and SrCl_2_·6H_2_O (0.1 mmol) in MeCN (4 mL) was stirred at room temperature for 3-10 h (Table 1). After the completion of the reaction, the mixture was diluted with water (5 mL), extracted with EtOAc (3 × 5 mL). The combined organic layer was washed with saturated aqueous sodium bicarbonate solution (3 mL), dried over anhydrous Na_2_SO_4_ and concentrated in vacuo. The residue was purified by column chromatography (silica gel, hexane-EtOAc) to afford pure *gem*-dihydroperoxides **2** or hydroxyl-hydroperoxides **4 **(Table 1). The products were characterized on the basis of their physical properties and spectral (^1^H-, ^13^C-NMR and MS) analyses and compared with literature data [32,33,37,40,41]. The spectral (^1^H-, ^13^C-NMR and MS) data of some representative products are given below.

*Undecane-2,2-dihydroperoxide* (**2a**) [32]. ^1^H-NMR (200 MHz, CDCl_3_): δ 9.51 (br s, 2H), 1.76–1.60 (m, 2H), 1.38 (s, 3H), 1.32–1.19 (br s,14H), 0.82 (t, *J* = 7 Hz, 3H); ^13^C-NMR (50 MHz, CDCl_3_): 112.3, 33.4, 32.0, 29.4, 29.1, 28.4, 23.6, 22.5, 17.6, 13.8, 13.5; FABMS: *m/z* 243 [M+Na]^+^.

*4-Methylpentane-2,2-dihydroperoxide* (**2d**) [32]. ^1^H-NMR (200 MHz, CDCl_3_): δ 9.54 (br s, 2H), 1.80 (m, 1H), 1.62 (d, *J* = 7 Hz, 2H), 1.42 (s, 3H), 0.98 (d, *J* = 7 Hz, 6H); FABMS: *m/z* 173 [M+Na]^+^.

*Cyclohexane-1,1-dihydroperoxide* (**2e**) [37]. ^1^H-NMR (200 MHz, CDCl_3_): δ 9.70 (br s, 2H), 1.93–1.70 (m, 4H), 1.67–1.39 (m, 6H); FABMS: *m/z* 171 [M+Na]^+^.

*Methy-phenyl-1,1-dihydroperoxide* (**2h**) [33]. ^1^H-NMR (200 MHz, CDCl_3_): δ 9.16 (br s, 2H), 7.50–7.43 (m, 2H), 7.38–7.26 (m, 3H), 1.69 (s, 3H); FABMS: *m/z* 193 [M+Na]^+^.

*Phenylmethylene-1,1-dihydroperoxide* (**2l**) [33]. ^1^H-NMR (200 MHz, CDCl_3_): δ 9.57 (br s, 2H), 7.42–7.28 (m, 5H), 6.24 (s, 1H); FABMS: *m/z* 179 [M+Na]^+^.

*(4-Methylphenyl)methylene-1,1-dihydroperoxide* (**2m**) [41]. ^1^H-NMR (200 MHz, CDCl_3_): δ 9.71 (br s, 2H), 7.30 (d, *J* = 8 Hz, 2H), 7.15 (d, *J* = 8 Hz, 2H), 6.28 (s,1H), 2.32 (s, 3H); ^13^C-NMR (50 MHz, CDCl_3_): 139.5, 129.4, 129.0, 126.7, 109.8, 21.1; FABMS: *m/z* 193 [M+Na]^+^.

*(4-Chlorophenyl)methylene-1,1-dihydroperoxide* (**2n)** [32]. ^1^H-NMR (200 MHz, CDCl_3_): δ 9.94 (br s, 2H), 7.85–7.34 (m, 4H), 6.26 (s, 1H); ^13^C-NMR (50 MHz, CDCl_3_): 139.6, 129.4, 129.0, 126.8, 10.02; FABMS: *m/z* 213 [M+Na]^+^.

*(4-Cyanophenyl)methylene-1,1-dihydroperoxide* (**2p)**. White solid; m.p. 107–110 ºC; IR (KBr): 3,414, 2,916, 2,235, 1,611, 1,405, 1,333, 1,243, 1,199, 1,122, 1,083, 977, 824 cm^-1^; ^1^H-NMR (200 MHz, CDCl_3_): δ 10.08 (s, 2H), 8.04–7.78 (m, 4H), 7.24 (s, 1H); ^13^C-NMR (50 MHz, CDCl_3_): δ 139.3, 129.4, 128.0, 126.1, 117.0, 112.1; FABMS: *m/z* 204 [M+Na]^+^; Anal. Calcd for C_8_H_7_NO_4_: C, 53.04; H, 3.86; N, 7.73. Found: C, 53.15; H, 3.98; N, 7.78.

*Octane-1,1-hydroxyhydroperoxide* (**4q**) [42]. Colorless oil; IR (KBr): 3,374, 3,028, 2,931, 2,863, 1,496, 1,454, 1,357, 1,242, 1,078, 1,030, 924, 748, 699 cm^-1^; ^1^H-NMR (200 MHz, CDCl_3_): δ 8.20 (br s, 1H), 7.00 (br s, 1H), 4.90 (t, *J* = 7 Hz, 1H), 2.10–0.70 (m, 15H); ^13^C-NMR (50 MHz, CDCl_3_): δ 101.2, 32.6, 30.0, 28.5, 24.0, 20.1, 14.0; FABMS: *m/z* 185 [M+Na]^+^.

*3-Phenylpropane-1,1-hydroxyhydroperoxide* (**4r**) [42]. Colorless oil; IR (KBr): 3384, 3062, 3027, 2902, 2861, 1496, 1457, 1376, 1242, 1079, 1031, 923, 747, 700 cm^-1^; ^1^H NMR (200 MHz, CDCl_3_): δ 9.78 (br s, 1H), 8.65 (br s, 1H), 7.60–7.00 (m, 5H), 5.10 (t, *J* = 7 Hz, 1H), 2.60 (t, *J* = 8 Hz, 2H), 2.15–1.60 (m, 2H); ^13^C NMR (50 MHz, CDCl_3_): δ 141.5, 127.5, 125.0, 100.0, 32.2, 28.5; FABMS: *m/z* 191 [M+Na]^+^.

## 4. Conclusions

In summary, a new efficient homogeneous catalyst SrCl_2_·6H_2_O has been shown to promote the synthesis of *gem*-dihydroperoxides from aliphatic and aromatic ketones and aldehydes using aqueous H_2_O_2_ (30%) in acetonitrile at room temperature. The attractive features of this new approach are the readily available and non-expensive catalyst, high yields of the products, mild reaction conditions and the operational simplicity of the procedure.

## References

[1] Zmitek K., Zupan M., Iskra J. (2007). Synthetic strategies for a biologically important class of gem-dihydroperoxide and perketal derivatives. Org. Biomol. Chem..

[2] Iskra J., Bonnet-Delpon D., Begue J.P. (2003). One-pot synthesis of non-symmetric tetraoxanes with the H_2_O_2_/MTO/fluorous alcohol system. Tetrahedron Lett..

[3] Terent’ev A.O., Kutkin A.V., Starikova Z.A., Antipia M.Y., Ogibin Y.N., Nikishina G.I. (2004). New preparation of 1,2,4,5-tetraoxanes. Synthesis.

[4] Ito T., Tokuyasu T., Masuyama A., Nojima M., McCullough K.J. (2003). Synthesis of novel macrocyclic peroxides by bis(*sym*-collidine)iodine (I) hexafluorophosphate-mediated cyclization of unsaturated hydroperoxides and unsaturated alcohol. Tetrahedron.

[5] Zmitek K., Stavber S., Zupan M., Bonnet-Delpon D., Iskra J. (2006). Fluorinated alcohol directed formation of dispiro-1,2,4,5-tetraoxanes by hydrogen peroxide under acid conditions. Tetrahedron.

[6] Dong Y.X., Vennerstrom J.L. (1998). Dispiro-1,2,4,5-tetraoxanes via ozonolysis of cycloalkanone *O*-methyl oximes: a comparison with the peroxidation of cycloalkanones in acetonitrile sulfuric acid media. J. Org. Chem..

[7] Zmitek K., Stavber S., Zupan M., Bonnet-Delpon D., Charneau S., Grellier P., Iskra J. (2006). Synthesis and antimalarial activities of novel 3,3,6,6-tetraalkyl-1,2,4,5-tetraoxanes. J. Bioorg. Med. Chem..

[8] Opsenica D., Pocsfalvi G., Juranic Z., Tinant B., Declercq J.P., Kyle D.E., Milhous W.K., Solaja B.A. (2000). Cholic acid derivatives as 1,2,4,5-tetraoxane carriers: structure, antimalarial and antiproliferative activity. J. Med. Chem..

[9] Dong Y. (2002). Synthesis and antimalarial activity of 1,2,4,5-tetraoxanes. Mini-Rev. Med. Chem..

[10] Terent’ev A.O., Platonov M.M., Tursina A.I., Chemyshev V.V., Nikishin G.I. (2008). Synthesis of cyclic peroxides containing the Si-*gem*-bisperoxide fragment 1,2,4,5,7,8-hexaoxa-3-silonanes as a new class of peroxides. J. Org. Chem..

[11] Ghorai P., Dussault P.H., Hu C. (2008). Synthesis of spiro-bisperoxyketals. Org. Lett..

[12] Kim H.S., Nagai Y., Ono K., Begum K., Wataya Y., Hamada Y., Tsuchiya K., Masuyama A., Nojima M., McCullough K.J. (2001). Synthesis and antimalarial activity of novel medium-sized 1,2,4,5-tetraoxacycloalkanes. J. Med. Chem..

[13] Jakka K., Liu J., Zhao C.G. (2007). Facile epoxidation of α,β-unsaturated ketones with cyclohexylidenebishydroperoxide. Tetrahedron Lett..

[14] Saneyyoshi H., Miyata K., Seio K., Sekine M. (2006). 1,1-Dihydroperoxycyclododecane as a new, crystalline non-hygroscopic oxidizer for the chemical synthesis of oligodeoxyribonucleotides. Tetrahedron Lett..

[15] Jon Paul Selvam J., Suresh V., Rajesh K., Chanti Babu D., Suryakiran N., Venkateswarlu Y. (2008). A novel rapid sulfoxidation of sulfides with cyclohexylidenebishydroperoxide. Tetrahedron Lett..

[16] Bunge A., Hamann H.J., McCalmont E., Leibscher J. (2009). Enantioselective epoxidation of 2-substituted 1,4-naphthoquinones using *gem*-dihydroperoxides. Tetrahedron Lett..

[17] Adam W. (2000). Peroxide Chemistry: Mechanistic And Preparative Aspects Of Oxygen Transfer.

[18] Ando W. (1992). Organic Peroxides.

[19] Tang Y.Q., Dong Y.X., Vennerstrom J.L. (2004). Synthetic peroxides as antimalarials. Med. Res. Rev..

[20] Masuyama A., Wu J.M., Nojima M., Kim H.S., Wataya Y. (2005). 1,2,4,5-Tetraoxacycloalkanes: synthesis and antimalarial activity. Mini-Rev. Med. Chem..

[21] Borstnik K., Mpaik I.H., Shapiro T.A., Posner G.H. (2002). Antimalarial chemotherapeutic peroxides: artemisinin, yingzhaosu A and related compounds. Int. J. Parasitol..

[22] Wiesner J., Ortmann R., Jomaa H., Schlitzer M. (2003). New antimalarial drugs. Angew. Chem. Int. Ed..

[23] Hamada Y., Tokuhara H., Masuyama A., Nojima M., Kim H.S., Ono K., Ogura N., Wataya Y. (2002). Synthesis and notable antimalarial activity of acyclic peroxides, L-(alkyldioxy)-L-(methyldioxy)cyclododecanes. J. Med. Chem..

[24] Kim H.S., Tsuchiya K., Shibata Y., Wataya Y., Ushigoe Y., Masuyama A., Nojima M., McCullough K.J. (1999). Synthetic methods for unsymmetrically-substituted 1,2,4,5-tetroxanes and of 1,2,4,5,7-pentoxocanes. J. Chem. Soc., Perkin Trans..

[25] Tsuchiya K., Hamada Y., Masuyama A., Nojima M. (1999). Synthesis, crystal structure and anti-malarialactivity of novel spiro-1,2,4,5-tetraoxacycloalkanes. Tetrahedron Lett..

[26] Dong Y., Matile H., Chollet J., Kaminsky R., Wood J.K., Vennerstrom J.L. (1999). Synthesis and antimalarial activity of eleven dispiro-1,2,4,5-tetraoxane analogs of WR 148999. 7,8,15,16-tetraoxadispiro[5.2.5.2]hexadecanes substituted at the 1 and 10 positions with unsaturated and polar functional groups. J. Med. Chem..

[27] Ledaal T., Solbjor T. (1967). 1,1-Dihydroperoxycyclododecane. Acta Chem. Scand..

[28] Terent’ev A.O., Platonov M.M., Ogibin Y.N., Nikishin G.I. (2007). Convenient synthesis of geminal bishydroperoxides by the reaction of ketones with hydrogen peroxide. Synth. Cmmun..

[29] Jefford C.W., Li W., Jaber A., Boukouvalas J. (1990). A new method for the synthesis of *gem*-dihydroperoxides. Synth. Cmmun..

[30] Terent’ev A.O., Kutkin A.V., Platonov M.M., Ogibin Y.N., Nikishin G.I. (2003). A new method for the synthesis of bishydroperoxides based on a reaction of ketals with hydrogen peroxide catalyzed by boron trifluoride complexe. Tetrahedron Lett..

[31] Bunge A., Hamann H.J., Liebscher J. (2009). A simple, efficient and versatile synthesis of primary gem-dihydroperoxides from aldehydes and hydrogen peroxide. Tetrahedron Lett..

[32] Das B., Krishnaiah M., Veeranjaneyulu B., Ravikanth B. (2007). A simple and efficient synthesis of *gem*-dihydroperoxides from ketones using aqueous hydrogen peroxide and catalytic ceric ammonium nitrate. Tetrahedron Lett..

[33] Zmitek K., Zupan M., Stavber S., Iskra J. (2006). Iodine as a catalyst for efficient conversion of ketones to *gem*-dihydroperoxides by aqueous hydrogen peroxide. Org. Lett..

[34] Ghorai P., Dussault P.H. (2008). Mild and efficient Re(VII)-catalyzed synthesis of 1,1-dihydroperoxides. Org. Lett..

[35] Ghorai P., Dussault P.H. (2009). Broadly applicable synthesis of 1,2,4,5-tetraoxanes. Org. Lett..

[36] Ledaal T., Solbjor T. (1967). 1,1-Dihydroperoxycyclododecane. Acta Chem. Scand..

[37] Das B., Veeranjaneyulu B., Krishnaiah M., Balasubramanyam P. (2008). Synthesis of *gem*-dihydroperoxides from ketones using silica-supported sodium hydrogen sulfate as a heterogeneous catalyst. J. Mol. Catal. A.

[38] Ramirez A., Woerpel K.A. (2005). Synthesis of 1,2-dioxolanes by annulation reactions of peroxzcarbenium ions with alkenes. Org. Lett..

[39] Terent’ev A.O., Kutkin A.V., Troizky N.A., Ogibin Y.N., Nikishin G.I. (2005). Synthesis of geminal bisperoxides by acid-catalyzed reaction of acetals and enol ethers with *tert*-butyl hydroperoxide. Synthesis.

[40] Rieche A. (1931). Über oxyalkyl-hydroperoxyde. (VII. Mitteil. über alkylperoxyde). Chem. Ber..

[41] Zmitek K., Zupan M., Stavber S., Iskra J. (2007). The effect of iodine on the peroxidation of carbonyl compounds. J. Org. Chem..

[42] McClelland R.A., Coe M. (1983). Structure-reactivity effects in the hydration of benzaldehydes. J. Am. Chem. Soc..

